# A longitudinal analysis of early lung function trajectory in survivors of childhood Hodgkin lymphoma

**DOI:** 10.1002/cnr2.1661

**Published:** 2022-06-27

**Authors:** Katina Zheng, Mylène Bassal, Nicholas Mitsakakis, Tanita Cepalo, Jemila Seid Hamid, Franco Momoli, Joseph Reisman, Vimoj Nair, Dhenuka Radhakrishnan

**Affiliations:** ^1^ Faculty of Medicine University of Ottawa Ottawa Ontario Canada; ^2^ Department of Pediatrics, Division of Hematology/Oncology Children's Hospital of Eastern Ontario Ottawa Ontario Canada; ^3^ Children's Hospital of Eastern Ontario Research Institute Ottawa Ontario Canada; ^4^ Carleton University Ottawa Ontario Canada; ^5^ Ottawa Hospital Research Institute Ottawa Ontario Canada; ^6^ Department of Pediatrics, Division of Respirology Children's Hospital of Eastern Ontario Ottawa Ontario Canada; ^7^ Division of Radiation Oncology University of Ottawa Ottawa Ontario Canada; ^8^ ICES uOttawa Ottawa Ontario Canada

**Keywords:** cancer, chemotherapy, late effects, pulmonary function, radiation therapy

## Abstract

**Background:**

Childhood Hodgkin lymphoma survivors suffer from long‐term effects decades after treatment completion with a prevalence of pulmonary dysfunction of up to 65.2%.

**Aims:**

This study explored the early trajectory of pulmonary function in pediatric cancer patients with Hodgkin lymphoma who received pulmonary toxic therapy.

**Methods and Results:**

In this single‐center, 20‐year retrospective cohort study, we included patients who were <18 years old at diagnosis of Hodgkin lymphoma between January 1994 and December 2014, and received bleomycin or thoracic radiation. We measured pulmonary function and reported on percent predicted values for forced expiratory volume in 1 s, total lung capacity, and diffusing capacity of the lungs. We used linear mixed models to identify the association of clinical factors with longitudinal changes in lung function at time points before and after treatment completion. Of 80 children who met inclusion criteria, all were treated with bleomycin, and 83.8% received thoracic radiation. More than half (51.2%) of patients had any abnormalities in lung function measures during the study observation period which averaged 24.2 months (±31.1SD). Females, younger age at diagnosis and treatment with radiation were associated with lower lung function measurements at various time points. While the majority of children experienced a recovery of their lung function within 1–2 years after treatment completion, some children with these risk factors did not.

**Conclusion:**

Pulmonary function abnormalities begin early in children treated for Hodgkin lymphoma. While the majority of children demonstrate a slow and continuous improvement in lung function back to baseline over time, we recommend routine asymptomatic screening of pulmonary function in certain childhood cancer survivors, particularly females, those diagnosed young and patients who received radiation therapy.

## 1 INTRODUCTION

Over the last few decades, improved antineoplastic therapies have resulted in significant improvements in cancer survival rates. For childhood and adolescent cancers, the 5‐year survival has risen to over 85%^1^ creating a growing population of childhood cancer survivors (CCS). In 2015, over 360 000 CCS were living in the United States.^1^

Although treatment regimens, which can include radiation, chemotherapy, and surgery, are highly effective, they can also cause significant long‐term morbidity and mortality as these young survivors age.^2^ Secondary malignancies and organ dysfunction can develop just a few years after the successful cure of the primary cancer.^3^ The St Jude Lifetime Cohort Study found that by age 50, 96% of CCS will have had a severe, life threatening, or fatal chronic health condition, compared to 85% of community controls.^4^

Pulmonary dysfunction is the second leading cause of mortality in CCS,^5^ with survivors being 8.8 times more likely to die from a pulmonary event than the general population.^3^ It is also one of the most common morbidities of treatment, with a prevalence of 65% in adulthood.^6^ There are a small number of cross‐sectional studies that report on a range of mild obstructive and restrictive pulmonary function abnormalities observed in 41%–53% of CCS within the first few years after treatment completion.^7,^, ^8^ However, the exact timing of the onset of this pulmonary dysfunction is not well understood.^9^, ^10^ The increase in prevalence of pulmonary morbidity seen in later adulthood may be due to lung injury occurring during a period of lung development, leaving a deficit in growth potential, thereby amplifying the natural decline in lung function with age.^11^ Alternatively, the lungs may be more susceptible to the toxic effects of radiation and immunosuppression during this growth phase leading to ongoing damage even after completion of treatment. Common pulmonary toxic therapies, including bleomycin, as well as busulfan, lomustine, carmustine,^5^ and thoracic radiation, have been shown to result in sequelae including pulmonary fibrosis, and interstitial pneumonitis^12^ which can lead to progressive lung damage. Radiation can also result in oxidative stress, leading to long‐term tissue damage, and chronic inflammation.^13^, ^14^

Improved understanding of the onset of pulmonary dysfunction in CCS and its evolution over time can improve early detection and provide a window for early intervention of this potentially devastating complication in patients with risk factors. In this retrospective cohort study, we sought to better understand the early trajectory of lung function and the association with clinical factors in children who received pulmonary toxic cancer therapies for Hodgkin lymphoma (HL), one of the most prevalent childhood cancers.^15^

## 2 METHODS

### 2.1 Study design

We performed a retrospective cohort study of children who received pulmonary toxic chemotherapy with or without thoracic radiation for treatment of HL over a 20‐year period and observed lung function up to 5 years after the completion of cancer treatment. This study is reported in accordance with the STROBE statement.^16^

### 2.2 Participants and setting

We included all patients who were 6 to <18 years of age at diagnosis of HL and treated at the Children's Hospital of Eastern Ontario (CHEO) between January 1994 and December 2014 for a diagnosis of HL. CHEO is a tertiary care pediatric hospital in Ottawa, Canada with a medium‐sized pediatric oncology program, treating approximately 70 new oncology patients per year. Included patients received pulmonary toxic treatment and underwent pulmonary function testing. We limited inclusion to children with a minimum of two acceptable pulmonary function tests (PFT), to allow a determination of change over time, with at least one test performed during the treatment period and one within 90 days following treatment completion and prior to any relapse, in order to capture the onset of pulmonary function changes related to initial treatment. Children were followed for a minimum of 2 years and up to 5 years post treatment completion. Our center performs pulmonary function testing only on children 6 and older, so children below this age cut‐off were excluded.

### 2.3 Pulmonary function parameters

The main lung function parameter studied was percent predicted (pp) forced expiratory volume in 1 s (FEV_1_) as measured using spirometry. Additional lung function parameters included in analysis were total lung capacity (TLC), measured using body plethysmography, and diffusion capacity of the lungs for carbon monoxide (DLCO), which was adjusted for the patient's most recent hemoglobin level. Trained respiratory therapists performed and independently interpreted all PFTs for acceptability and reproducibility at the time of test completion in accordance with American Thoracic Society standards.^17^ Pulmonary function was measured with the *Vmax*® *Encore PFT system* (Care Fusion, Yorba Linda, California). A modified version of the Morris et al. reference equations^18^ which adjust for patient age, sex and height and are well suited to the local study population, were used to determine pp values. There may have been some delays between HL diagnosis and treatment start date for some patients. As such, data from all available PFTs performed as early as 30 days pre‐treatment initiation and up to the end of each patient's follow up at CHEO were collected retrospectively from medical records.

As per routine care practices at CHEO, all children who received pulmonary toxic therapy underwent pulmonary function testing at the end of treatment regardless of the presence of symptoms. For patients with one or more recurrences of their primary cancer, all treatment and pulmonary function data beyond the date of the relapse was censored and excluded from the analysis. This ensured that analysis was limited to lung function changes solely due to initial pulmonary toxic exposure and to avoid any confounding effects of cumulative treatment exposures due to relapse. Children who died were similarly censored. Given the retrospective nature of the study, there were some variations between patients in the exact timing of lung function testing relative to treatment start date or completion date as these tests were performed in coordination with treatments or follow up clinic visits.

### 2.4 Primary exposure: Pulmonary toxic therapy

Agents with significant pulmonary toxic properties identified from the literature include pulmonary radiation and select chemotherapy agents (e.g., bleomycin) and exposure to each of these was collected from patient records. For the purposes of descriptive analysis only, we stratified thoracic radiation into high volume or low volume depending on the volume of lung included in the radiation field. Mantle, mediastinum, and whole lung irradiation were considered high‐volume thoracic radiation exposure, while neck, abdominal, inverted Y, para‐aortic, or supraclavicular radiation given in the absence of high volume radiation were considered low volume thoracic radiation exposure. Radiation exposure was dichotomized (yes/no) for adjusted analyses. Internationally approved standardized pediatric oncology treatment protocols based on cancer type and extent of disease were used for both chemotherapy and radiation therapy. These have only modestly changed throughout the study time‐period.

### 2.5 Additional variables

Variables examined in this study also included patient demographics (age, height, weight, sex, comorbidities), cancer characteristics (sites of disease, relapse history), and treatment history (chemotherapy agents, radiation fields, or thoracic surgery). Side effects of therapy with the potential to affect measured pulmonary function such as pneumonitis, and respiratory related acute care visits (i.e., hospital inpatient admissions and emergency department visits) were also captured. Data were collected retrospectively from medical records at CHEO.

### 2.6 Statistical analysis

Descriptive statistics were summarized for the primary and secondary outcomes as well as demographics, therapeutic exposures, hospitalizations and other clinical variables. Mean and standard deviation (SD) were used for continuous outcomes and binary outcomes were summarized using frequency and proportions, expressed as percentage (%).

The visit date for the last recorded cancer treatment (radiation or chemotherapy) was defined as time zero. Data were summarized into intervals before (phase one) and after treatment completion, with the post‐treatment time‐period further subdivided into <1 year post treatment (phase two), and ≥1 year post‐treatment (phase three). These periods were defined after careful consideration of the distribution of the primary and secondary outcomes and the corresponding time trajectories.

We performed graphical descriptive analysis for all three pulmonary function outcomes (FEV_1_, TLC, and DLCO) in order to visualize similarities and differences in lung function trajectories from the time of diagnosis and after cancer treatment. A series of linear mixed models with random intercept were fitted for longitudinal analysis and evaluation of lung function change (i.e., slope) within each phase. *p*‐values of <.05 indicated a significant change in average lung function within each phase, with the estimate indicating whether this was a positive (i.e., rise) or negative change (i.e., decrease). Time was used as a piecewise linear predictor for the models, with its effect changing at two time points, end of treatment and one‐year post‐treatment. The linear mixed model approach allowed adjustment for small variations in the timing of PFT for individual patients. Other covariates used in the models were sex (reference = females), age at diagnosis (reference = 15 years) and radiation treatment (reference = no radiation exposure). The interaction of time with these covariates was considered. Any extreme outliers which were likely to represent invalid entries were removed. All analysis was performed using the R statistical software package.^19^

## 3 RESULTS

Over the 20‐year study period from January 1994 to December 2014, 97 children had a diagnosis of HL that was treated at CHEO. Of these, 2 were <6 years old at diagnosis, 10 did not receive pulmonary toxic therapies and 5 performed fewer than 2 acceptable PFTs. As such, 80 children met inclusion criteria for this study with both a diagnosis of HL and data available for pulmonary function testing. The only pulmonary toxic therapies received by included patients during their initial treatment (excluding relapses) were bleomycin and radiation.

The median age at diagnosis for included patients was 14.1 ± 2.9 (SD) years. In total, 8.8% had evidence of malignancy in the lungs, but none underwent thoracic surgery. During treatment, 22.5% had a respiratory complication that resulted in either an emergency department visit or inpatient admission, compared to 5% post‐treatment. During follow‐up, approximately 12.5% and 18.8% reported ever smoking tobacco or marijuana respectively, and 12.5% were ever diagnosed with asthma. Thirteen (15.1%) children relapsed following their initial treatment, and five (5.8%) patients died before the end of the study period.

The mean treatment duration was 199 ± 113 (SD) days with a median of 163 days. The mean number of PFTs completed per patient was 5.2 ± 3.0 (SD), and 34 patients had PFT's performed within all three phases. The average length of follow up was 24.2 ± 31.1 (SD) months from the date of diagnosis to the last PFT at CHEO prior to age 18. Up to 46.2% of patients had an FEV_1_ less than 80 pp at any time point, 11.2% ever had a TLC less than 80 pp and 51.2% of children ever had a DLCO less than 80 pp. Further characteristics of the study population are in Table 1, and raw plots of pulmonary function over time are in Figures [Link], [Link].

**TABLE 1:** Study cohort characteristics

Characteristic	Total *N* = 80
Age, mean (SD)	14.1 (2.9)
Female sex, *n* (%)	31 (38.8)
Age at treatment completion, mean (SD)	14.7 (2.9)
Body mass index, mean (SD)	21.2 (4.6)
Cancer location, *n* (%)	
Mediastinum	56 (70.0)
Pulmonary	7 (8.8)
Abdomen	9 (11.2)
Neck	48 (60.0)
Other	39 (48.8)
Treatment duration, mean days (SD)	199.7 (113.5)
Received pulmonary toxic chemotherapy, *n* (%)	80 (100.0)
Received radiation therapy, *n* (%)	67 (83.3)
Low volume	34 (42.5)
High volume	33 (41.2)
Reported smoking, *n* (%)	
Current	12 (15.0)
Past	3 (3.8)
Never	41 (51.2)
Unknown	24 (30.0)
Asthma, *n* (%)	10 (12.5)
Number of pulmonary function tests, mean (SD)	5.2 (3.0)
Respiratory related acute care visits, *n* (%)	18 (22.5)
Relapsed, *n* (%)	13 (15.1)
Died *n*, (%)	5 (5.8)

*Note*: All values represent at time of cancer diagnosis, except where indicated. SD = standard deviation. Low Thoracic Radiation Volume = neck, abdominal, inverted Y, para‐aortic, flank or supraclavicular radiation; High Thoracic Radiation volume = Mantle, mediastinum, whole lung and total body irradiation.

**FIGURE 1:**
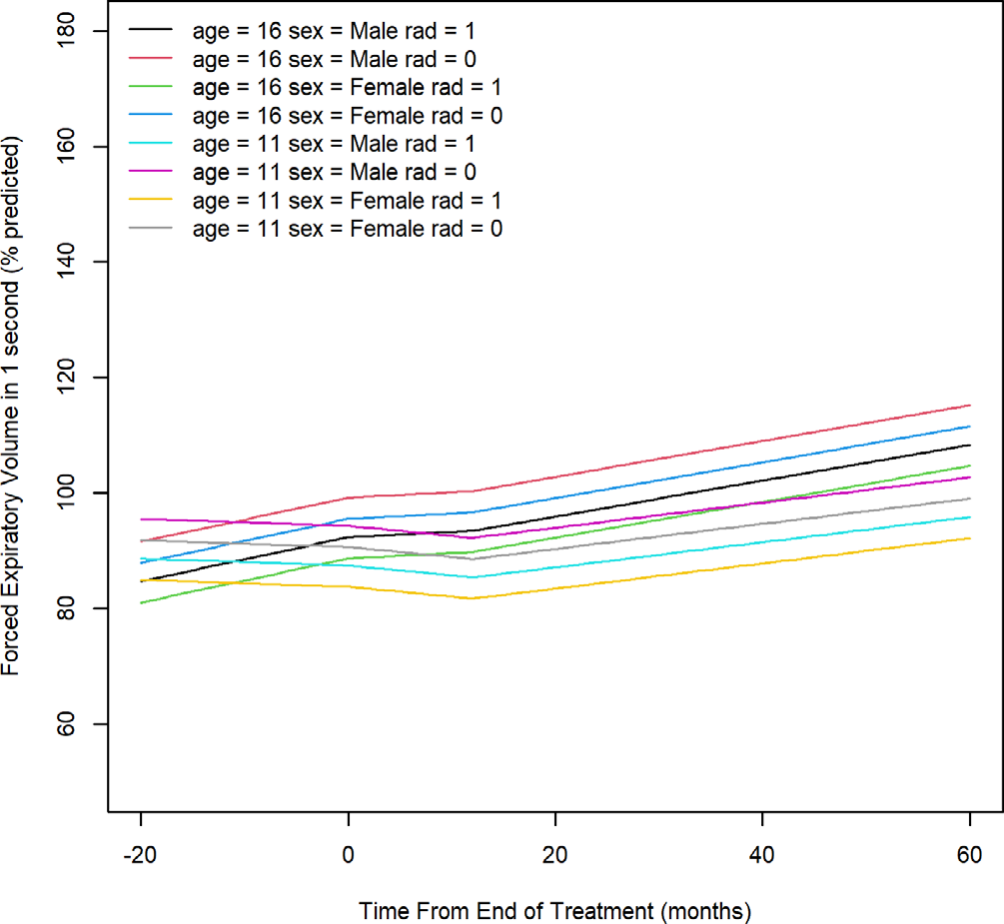
Trajectory of forced expiratory volume in 1 s over timeage 16 = 16 years old; age 11 = 11 years old; rad = radiation therapy; 0 = not received, 1 = received. The left limit of the X‐axis represents the earliest treatment start date for any patient. Value zero on the X‐axis represents the end of treatment completion for all patients

For the outcome FEV_1_, we observed an interaction between time and age of diagnosis. (Table S1) While the plots suggest a rising trend in FEV_1_ during phase 1 for older children, and a drop in FEV_1_ for younger children in phase 1 and 2, (Figure 1) these differences were not significant. For older children (≥15 years) there was a significant rise in FEV_1_ during phase three by at least 0.29 pp/month (95% CI: 0.14–0.44 pp/month, *p* = .0002). Children who received radiation had on average a 6.87 pp (95% CI: 1.1–12.7 pp, *p* = .02) lower FEV_1_ compared to children who did not receive radiation in all phases. Males appeared to have higher FEV_1_ values during all observation phases, though this was not statistically significant in our model (*p* = .11).

**FIGURE 2:**
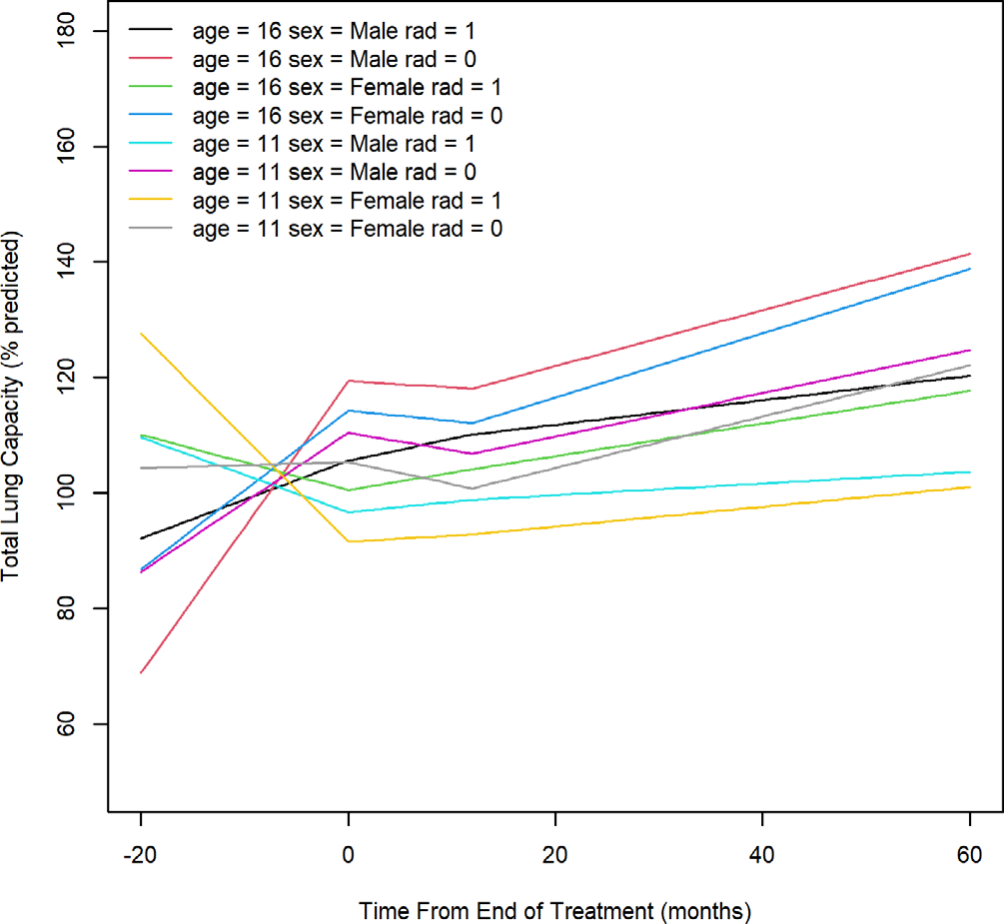
Trajectory of Total lung capacity over timeage 16 = 16 years old; age 11 = 11 years old; rad = radiation therapy; 0 = not received, 1 = received. The left limit of the X‐axis represents the earliest treatment start date for any patient. Value zero on the X‐axis represents the end of treatment completion for all patients

For the outcome of TLC, an interaction between age at diagnosis, sex and radiation exposure was identified during the different observation phases (Table S2). In examination of the plots (Figure 2), in older males as well as in older females without radiation exposure, there appeared to be an increase in TLC during phase one, followed by a slight drop in phase two. TLC then was observed to recover, and increase during phase three. Similar patterns were noted in younger males who had not received radiation. Older females (i.e., ≥15 years) with radiation exposure demonstrated a drop in TLC during phase one, followed by a steady significant increase of 0.53 pp/month (95% CI: 0.23–0.84 pp/month, *p* = .002) in phase three. TLC appeared to drop during phase one in younger age females and males who received radiation and suggested a slow increase following the end of treatment in phase two and three, but did not recover to start of treatment, baseline lung function values.

**FIGURE 3:**
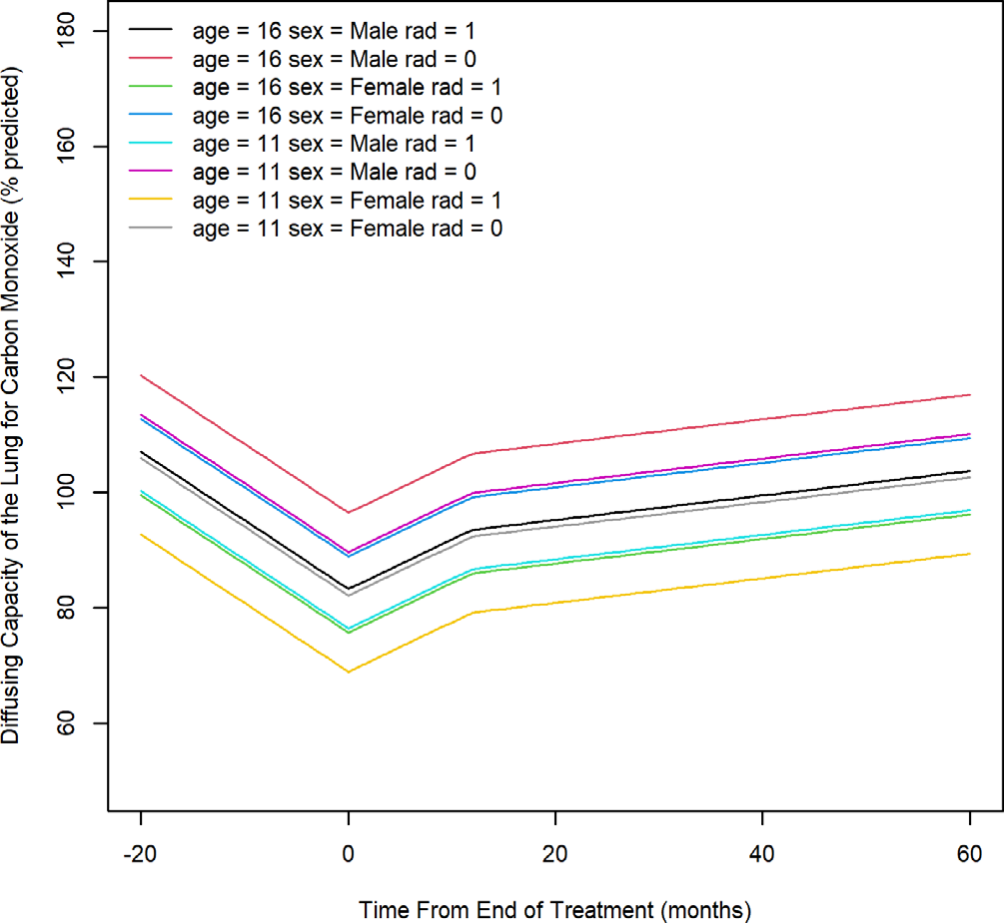
Trajectory of diffusing capacity of the lung for carbon monoxide over timeage 16 = 16 years old; age 11 = 11 years old; rad = radiation therapy; 0 = not received, 1 = received. The left limit of the X‐axis represents the earliest treatment start date for any patient. Value zero on the X‐axis represents the end of treatment completion for all patients

For the outcome of DLCO, for all children there was a significant decline of 1.18 pp/month (95% CI: 0.64–1.73 pp/month, *p* < .0001) during phase one, followed by a rapid increase of 0.85 pp/month during phase two (95% CI: 0.51–1.2 pp/month, *p* < .0001), and a slower but continued increase in DLCO of 0.21 pp/month (95% CI:0.09–0.34 pp/month; *p* = .001) during phase three (Figure 3, Table S3). Across all time points, older children demonstrated significantly higher values of DLCO (*p* = .003), and males generally had a DLCO that was 7.55 pp (95% CI: 2.41–12.7 pp, *p* = .001) higher than in females. The DLCO for children who received radiation across all observation phases was 13.2 pp lower (95% CI: 6.78–19.7, *p* = .0001) than for those who did not receive radiation. Overall, the lowest DLCO was seen in younger female children who received radiation, and this difference persisted through the entire observation period.

## 4 DISCUSSION

This study is one of the first to explore the early trajectory of pediatric lung function following pulmonary toxic therapy using objective pulmonary function testing. Overall, we observed three general phases in pulmonary function change in a cohort of children treated for HL: 1) during treatment, 2) during the 1 year immediately following treatment completion, and 3) more than 1 year post treatment. While most children had minimal reductions in lung function and demonstrated recovery to their start of treatment, baseline lung function by phase three, some children, particularly those who were younger at diagnosis and who received radiation did not. In general, females demonstrated lower lung function than males during all observation time points.

We hypothesize that for the children who experienced an improvement in TLC during phase one, this may have resulted from a combination of cancer location and resolution of an acute illness. Mediastinal masses, which are commonly found in HL, can decrease pulmonary function due to airway compression and mass effect, and present with respiratory symptoms at diagnosis.^20^ We did not observe a significant reduction in FEV_1_ in our cohort, however, in previous studies, patients presenting with respiratory symptoms have been seen to have an FEV_1_ of up to 28% lower, compared to asymptomatic patients.^20^ The small sample size in our study may have precluded us from identifying this effect which was shown only as a trend in our plots.

For children who experienced a drop in TLC as well as the majority of children who experienced a drop in DLCO during phase one or two (during treatment or shortly after treatment completion), we suspect this may be related to stiffening of the chest wall or parenchymal inflammation due to radiation pneumonitis. Radiation pneumonitis is a well‐documented side effect of treatment, often occurring within the first 2–4 months post‐radiation therapy,^21^ and has been previously shown to cause decreases in DLCO.^22^ Acute drops in pulmonary function can also be seen with bleomycin‐induced pneumonitis, which occurs in up to 46% of patients, and can decrease both TLC and DLCO.^23^, ^24^

For all children, in phase three, there was a slow and continued improvement of all pulmonary function parameters with a recovery to baseline in the majority of cases. Some of this recovery may reflect ongoing improvements in overall health, energy, and the ability to provide a maximal effort during pulmonary function testing. However, we suspect there may be ongoing healing of the lung inflammation induced by pulmonary toxic therapy that continues well after cancer therapy completion and takes months to improve and resolve.

For FEV_1_, the rate of recovery in phase three was observed to most depend on the age of the patient at diagnosis with younger children showing a much slower rate of improvement compared to older children. The association of younger age with reduced lung function and increased pulmonary complications post cancer therapy has been demonstrated in other studies,^5^ where these effects are hypothesized to be secondary to toxic insults to less developmentally mature lungs, resulting in a more lasting effect and potential reduction in growth potential.

For TLC, the rate of lung function recovery in phase three was observed to depend on both radiation exposure and age. Radiation is a known potentiator of the pulmonary toxic effects of chemotherapeutic agents such as bleomycin^25^, ^26^, ^27^ resulting in greater rates of interstitial pneumonitis, fibrosis, and mortality. Sex was a less important risk factor for determining longer term lung volume outcomes. Females, regardless of age, demonstrated lower lung volumes compared to males in phase one and two, but had a more rapid increase in lung volume recovery, compared to their male counterparts in phase three suggesting “catch up” growth.

For DLCO, recovery in phase three was observed to depend on all three of age, sex and radiation exposure with additive effects. Although sex is not typically considered a factor to influence lung function among children undergoing cancer therapy, adult females have been shown to experience disproportionately negative effects of cancer treatments on their lung function and mortality compared to their male counterparts.^3^ As such, adolescent and adult females may be at greater risk of lung injury as a result of receiving similar radiation treatment protocols despite having smaller average lung volumes.^28^ Another consideration is the effect of puberty on lung function. Previous reports have shown that lung function measured by plethysmography and single breath gas transfer (TLC and DLCO respectively) in healthy children rise discontinuously during puberty for males and correlate with an increase in thoracic volume, but this increase is not seen in females.^29^ This sex difference has also been demonstrated during puberty when measuring FEV_1_.^30^ This natural difference in lung growth may contribute to the difference in lung function recovery from pulmonary toxic treatment during this important phase of development. Similar sex‐related differences in pulmonary function and poorer health outcomes among females after puberty have been shown in other lung diseases including asthma and cystic fibrosis.^31^, ^32^, ^33^

This is one of the few studies to perform a longitudinal exploration of the early trajectory of lung function in CCS who received pulmonary toxic therapies. This study uniquely observed that previously identified risk factors for poor lung function, specifically, being female, younger age at diagnosis, and radiation therapy, together demonstrated additive deleterious effects on lung health and function during three different phases of recovery, with many children only returning to baseline lung function values well past treatment completion. Studies in adult CCS demonstrate a high prevalence of respiratory abnormalities compared to the general population,^31,^, ^32,^, ^34^ and our study shows that these lung function abnormalities start very early and are most pronounced in a select population of patients.

There have been a small number of previous studies describing pulmonary function abnormalities in CCS. The two that are most comparable included children with HL who received bleomycin. Both of these studies were cross sectional in design and do not provide information on the time of onset of observed pulmonary function abnormalities as in our longitudinal study. Only one of these previous studies explored the association between lung function abnormalities and clinical risk factors and similarly found a higher prevalence of abnormalities among younger children.^9^, ^10^ Our study adds to the literature as it sheds light on three different observation phases where lung function abnormalities occur and demonstrates how different clinical risk factors influence these abnormalities that evolve over time. As such, our study provides a better understanding of the natural history of pulmonary morbidity in CCS, and suggests there may be time points for early recognition of abnormalities and intervention in consultation with pulmonary specialists among children with certain risk factors.

Our study has several strengths, including its longitudinal analysis and elimination of certain typical biases despite its observational design. For example, because it is a standard of care at our center, all children had lung function testing at the end of their cancer treatment, regardless of symptoms. This reduced the potential risk of bias caused by a disproportionately higher number of post treatment PFTs being performed in children with respiratory dysfunction. Similarly, our use of percent predicted lung function measures which are inherently referenced against a control population and are adjusted for height, age, and sex, negated the need for a separate control group.

The biggest limitation of this study is its retrospective, and observational design, which hindered our ability to accurately capture exposure to certain risk factors known to affect lung function. For example, despite routine assessment of tobacco and marijuana use in adult patients during clinical follow‐up, these exposures are not routinely recorded for children. As such, the timing of these exposures relative to change in lung function could not be correlated in this study. Nevertheless, we found smoking rates similar to the general pediatric population in Ontario, with previous studies reporting that 16% of Ontario students in grades 7–12 had smoked in the last year and 19% had used marijuana.^35^ The prevalence of asthma in our cohort was below the provincial prevalence in youth, which can be up to 25.5% in children 10–14 years,^36^ and suggests that this information may not have been accurately captured in patient charts, as treating clinicians may under‐recognize the prevalence of asthma among oncology patients.^27^ In any case, the small sample size of this study limited our ability to explore the impact of these exposures, as well as other clinical predictors such as tumor bulk, location, stage, and presence of respiratory or B‐symptoms at diagnosis on early lung function trajectory. One further limitation was the inclusion of only pediatric‐aged CCS in this cohort as the majority were transitioned to the adult long‐term follow‐up program just prior to turning 18. Therefore, our study does not provide a full picture of potential ongoing recovery of lung function into adulthood.

Despite its limitations, our study sheds light on important factors to consider in the follow‐up of CCS, given our observation of a higher risk for pulmonary function abnormalities particularly among females who were diagnosed earlier and received radiation therapy. Given that a small proportion of children do not recover to start of treatment, baseline lung function well post treatment completion, we recommend routine screening of pulmonary function for CCS with these risk factors, even in the absence of clinical symptoms. Early identification of abnormalities might then warrant consultation with pulmonary specialists to intervene (e.g., smoking cessation treatment, pulmonary rehabilitation, treatment of comorbid respiratory conditions) and potentially mitigate morbidity into adulthood; however, most reassuring is that the majority of children demonstrate a slow and continued improvement in lung function over time. This longitudinal analysis of early lung function trajectory in CCS provides several hypotheses upon which to design a future multi‐center prospective study to better understand the influence of potentially modifiable factors that influence lung function outcomes in this vulnerable population.

## AUTHOR CONTRIBUTIONS

**Katina Zheng:** Conceptualization (supporting); data curation (lead); formal analysis (supporting); methodology (supporting); project administration (supporting); writing – original draft (lead); writing – review and editing (equal). **Mylène Bassal:** Conceptualization (equal); data curation (supporting); funding acquisition (supporting); methodology (supporting); project administration (supporting); supervision (supporting); writing – review and editing (equal). **Nicholas Mitsakakis:** Conceptualization (supporting); data curation (supporting); formal analysis (lead); methodology (equal); writing – review and editing (equal). **Tanita Cepalo:** Conceptualization (supporting); data curation (equal); methodology (supporting); writing – review and editing (equal). **Jemila Seid Hamid:** Conceptualization (equal); data curation (supporting); formal analysis (equal); methodology (equal); writing – review and editing (supporting). **Franco Momoli:** Conceptualization (equal); formal analysis (equal); methodology (equal); supervision (supporting); writing – review and editing (supporting). **Joseph Reisman:** Conceptualization (supporting); funding acquisition (supporting); methodology (supporting); supervision (supporting); writing – review and editing (equal). **Vimoj Nair:** Conceptualization (supporting); data curation (supporting); methodology (supporting); writing – review and editing (supporting). **Dhenuka Radhakrishnan:** Conceptualization (lead); data curation (supporting); formal analysis (supporting); funding acquisition (lead); methodology (equal); project administration (lead); supervision (lead); writing – original draft (equal); writing – review and editing (equal).

## CONFLICT OF INTEREST

The authors have stated explicitly that there are no conflicts of interest in connection with this article.

## ETHICS STATEMENT

This study was approved by the Research Ethics Board of the Children's Hospital of Eastern Ontario (CHEO).

## Supporting information


**Figure S1**. Trajectory of forced expiratory volume in 1 second over time.

**Figure S2**. Trajectory of total lung capacity over time.

**Figure S3**. Trajectory of diffusing capacity of the lung for carbon monoxide over time.

**Table S1** Fixed effects longitudinal analysis of percent predicted forced expiratory volume in one second over three time periods: during treatment, <1 year post treatment, >1 year post treatment.
**Table S2**. Fixed effects longitudinal analysis of percent predicted total lung capacity over three time periods: during treatment, <1 year post treatment, >1 year post treatment.
**Table S3**. Fixed effects longitudinal analysis of percent predicted diffusing capacity for carbon monoxide adjusted for Hemoglobin over three time periods: during treatment, <1 year post treatment, >1 year post treatment.

## Data Availability

The data that support the findings of this study are available on request from the corresponding author. The data are not publicly available due to privacy or ethical restrictions.
