# Supplementary figures and images for: A longitudinal analysis of early lung function trajectory in survivors of childhood Hodgkin lymphoma
Source: Cancer Rep (Hoboken). 2022 Jun 27;6(1):e1661. doi: 10.1002/cnr2.1661 (PMC9875613; doi:10.1002/cnr2.1661)

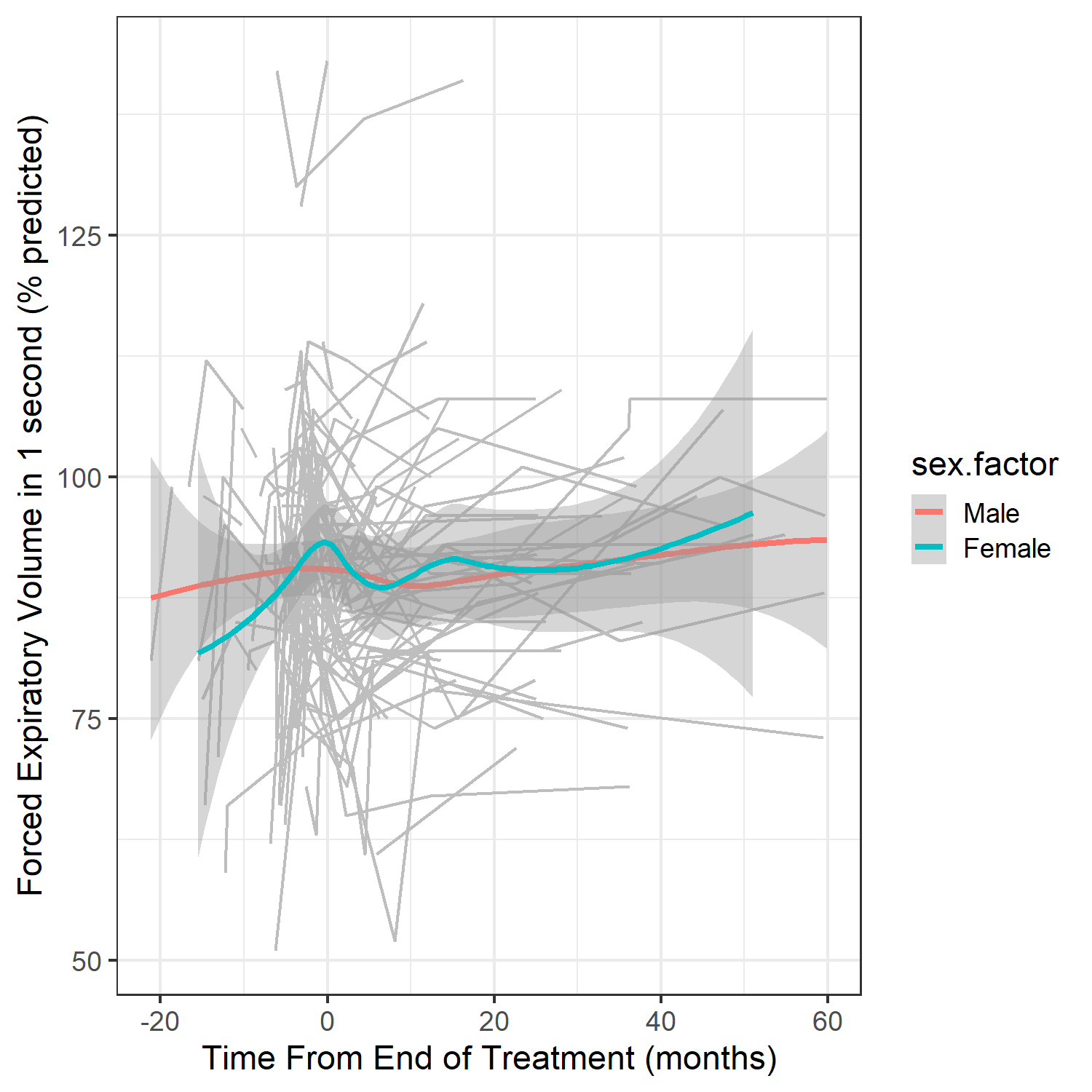

Supplement: Supplementary file 1 — Figure S1. Trajectory of forced expiratory volume in 1 second over time. [file CNR2-6-e1661-s003.tif]

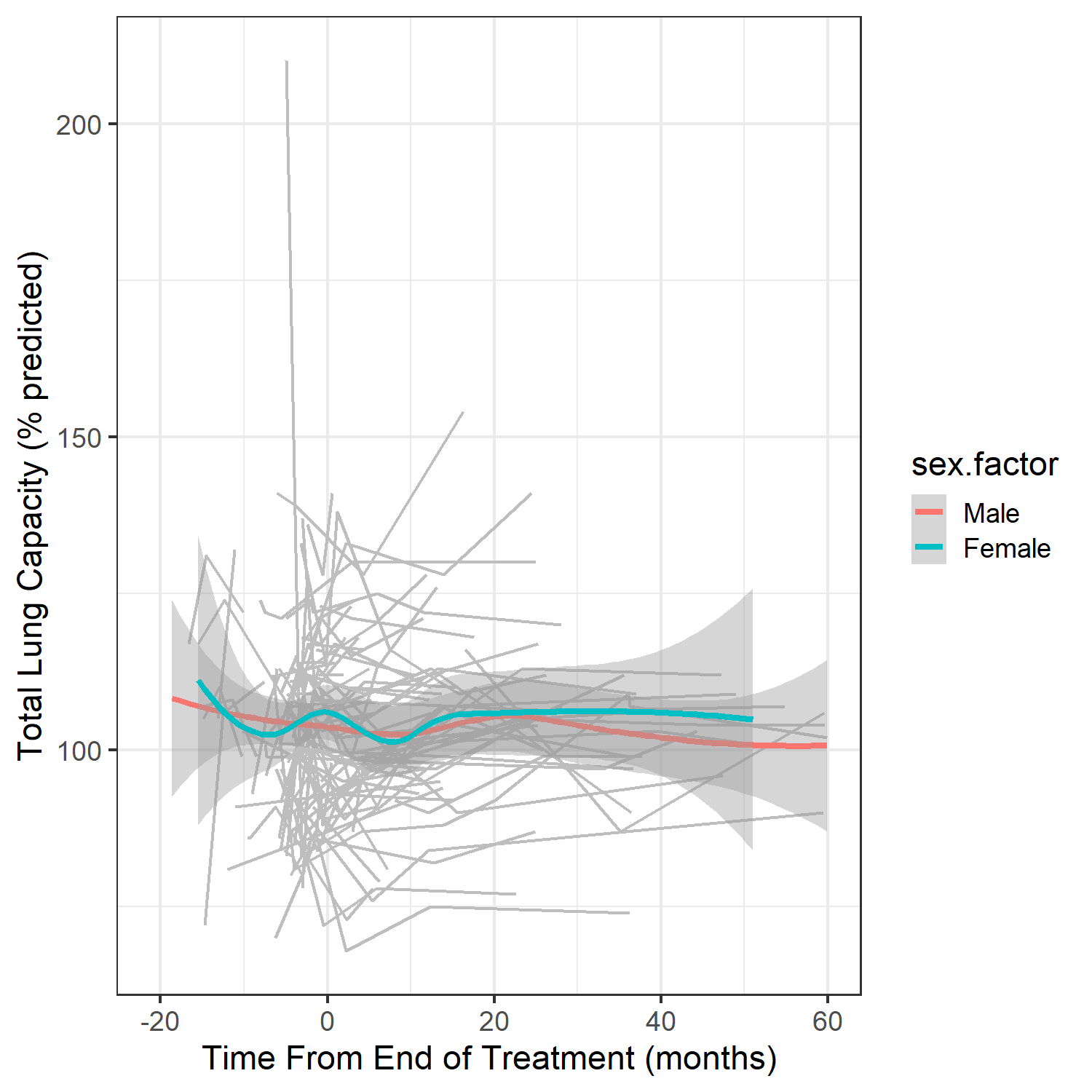

Supplement: Supplementary file 2 — Figure S2. Trajectory of total lung capacity over time. [file CNR2-6-e1661-s002.tif]

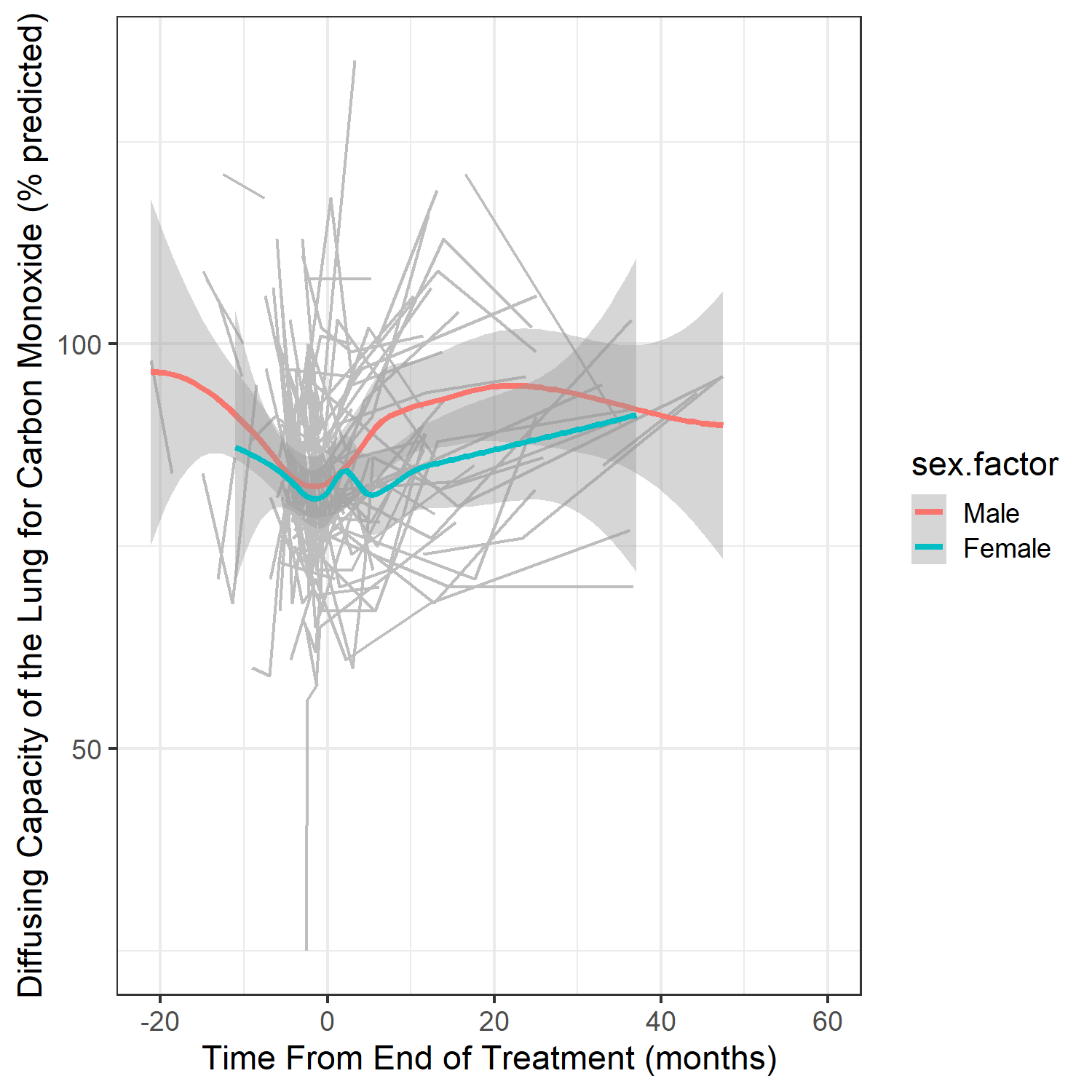

Supplement: Supplementary file 3 — Figure S3. Trajectory of diffusing capacity of the lung for carbon monoxide over time. [file CNR2-6-e1661-s001.tif]
