# Supplementary material for: A longitudinal analysis of early lung function trajectory in survivors of childhood Hodgkin lymphoma
Source: Cancer Rep (Hoboken). 2022 Jun 27;6(1):e1661. doi: 10.1002/cnr2.1661 (PMC9875613; doi:10.1002/cnr2.1661)
Supplement: Supplementary file 4 — Table S1 Fixed effects longitudinal analysis of percent predicted forced expiratory volume in one second over three time periods: during treatment, <1 year post treatment, >1 year post treatment. Table S2. Fixed effects longitudinal analysis of percent predicted total lung capacity over three time periods: during treatment, <1 year post treatment, >1 year post treatment. Table S3. Fixed effects longitudinal analysis of percent predicted diffusing capacity for carbon monoxide adjusted for Hemoglobin over three time periods: during treatment, <1 year post treatment, >1 year post treatment. [file CNR2-6-e1661-s004.docx]

**Table S1.** Fixed Effects Longitudinal Analysis of percent predicted forced expiratory volume in one second over three time periods: during treatment, <1 year post treatment, >1 year post treatment

|  | **Estimate** | **95% Lower CI** | **95% Upper CI** | **P value** |
| --- | --- | --- | --- | --- |
| **(intercept)** | 94.53 | 89.2 | 99.8 |  |
| **Phase1** | 0.29 | -0.16 | 0.76 | 0.22 |
| **Phase2** | 0.04 | -0.23 | 0.31 | 0.77 |
| **Phase3** | 0.29 | 0.14 | 0.44 | <0.01 |
| **Age** | 0.98 | -0.04 | 2.00 | 0.06 |
| **Male** | 3.67 | -0.75 | 8.09 | 0.11 |
| **Radiation** | -6.87 | -12.66 | -1.07 | 0.02 |
| **Phase1*age** | 0.09 | -0.07 | 0.24 | 0.28 |
| **Phase2*age** | 0.05 | -0.03 | 0.14 | 0.22 |
| **Phase3*age** | 0.02 | -0.003 | 0.04 | 0.10 |

phase 1 = time period during treatment, phase 2 = time period < 1 year post treatment, phase 3 = time period >1 year post treatment, age = age minus the reference age 15 years CI = confidence interval

*The model that includes the interaction with age is preferred

**Table S2.** Fixed Effects Longitudinal Analysis of percent predicted Total Lung Capacity over three time periods: during treatment, <1 year post treatment, >1 year post treatment

|  | **Estimate** | **95% Lower CI** | **95% Upper CI** | **P value** |
| --- | --- | --- | --- | --- |
| **(intercept)** | 112.48 | 104.32 | 120.64 | 0.00 |
| **Phase1** | 1.11 | -1.46 | 3.68 | 0.41 |
| **Phase2** | -0.22 | -0.95 | 0.51 | 0.56 |
| **Phase3** | 0.53 | 0.23 | 0.84 | <0.01 |
| **Age** | 1.79 | 0.57 | 3.01 | <0.01 |
| **Male** | 5.10 | -1.88 | 12.08 | 0.16 |
| **Radiation** | -13.77 | -22.80 | -4.74 | <0.01 |
| **Phase1*age** | 0.26 | 0.10 | 0.43 | <0.01 |
| **Phase1*male** | 1.15 | 0.22 | 2.10 | 0.02 |
| **Phase1*radiation** | -1.85 | -4.50 | 0.80 | 0.18 |
| **Phase2*age** | 0.04 | -0.05 | 0.13 | 0.41 |
| **Phase2*male** | 0.08 | -0.51 | 0.66 | 0.80 |
| **Phase2*radiation** | 0.48 | -0.32 | 1.29 | 0.25 |
| **Phase3*age** | 0.22 | -0.01 | 0.05 | 0.16 |
| **Phase3*male** | -0.07 | -0.33 | 0.19 | 0.60 |
| **Phase3*radiation** | -0.27 | -0.46 | -0.08 | <0.01 |

phase 1 = time period during treatment, phase 2 = time period < 1 year post treatment, phase 3 = time period >1 year post treatment, age = age minus the reference age 15 years, CI = confidence interval

*The model that includes the interactions with age, sex and radiation is preferred.

**Table S3.** Fixed Effects Longitudinal Analysis of percent predicted Diffusing Capacity for Carbon Monoxide adjusted for Hemoglobin over three time periods: during treatment, <1 year post treatment, >1 year post treatment

|  | **Estimate** | **95% Lower CI** | **95% Upper CI** | **P value** |
| --- | --- | --- | --- | --- |
| **(intercept)** | 87.56 | 87.71 | 93.42 | 0.00 |
| **Phase1** | -1.19 | -1.74 | -0.64 | <0.01 |
| **Phase2** | 0.85 | 0.51 | 1.20 | <0.01 |
| **Phase3** | 0.21 | 0.09 | 0.34 | <0.01 |
| **Age** | 1.36 | 0.50 | 2.23 | <0.01 |
| **Male** | 7.55 | 2.41 | 12.70 | <0.01 |
| **Radiation** | -13.22 | -19.67 | -6.78 | <0.01 |

phase 1 = time period during treatment, phase 2 = time period < 1 year post treatment, phase 3 = time period >1 year post treatment, age = age minus the reference age 15 years, CI = confidence interval

*The model with no interactions is preferred.
